# Methodological Innovations for Evidencing and Estimating Modern and Traditional Contraceptive Prevalence and Use Dynamics in Sub‐Saharan Africa

**DOI:** 10.1111/sifp.70054

**Published:** 2026-04-01

**Authors:** Nurudeen Alhassan, Nyovani Janet Madise, Jamaica Corker, Naa Dodua Dodoo, Ernestina Coast, F. Nii‐Amoo Dodoo, Jacques B. O. Emina, Saseendran Pallikadavath, Elizabeth Omoluabi, Maame B. Peterson, John A. Mushomi, Themba Mzembe, Eliya Msiyaphazi Zulu

## Abstract

Contraceptive prevalence estimates are indicators of the performance of family planning programs. Yet, available evidence suggests that national surveys may be underestimating the prevalence of traditional methods. The apparent underestimation of traditional methods stems from current approaches for collecting, analyzing, and reporting contraceptive data. We examined the effect of survey methodological innovations on the estimation of traditional and modern contraceptives. We used data from a cross‐country comparative study conducted in the Democratic Republic of Congo (DRC), Ghana, Kenya, and Nigeria. The sample comprised 9,075 in union and sexually active women not in union aged 15–49 years. The results showed that follow‐up method‐by‐method questioning increased the reporting of both traditional and modern methods, with the increase being much higher for modern methods, while reducing the percentage of nonusers. Revising the standard approach for computing contraceptive prevalence to account separately for concurrent traditional and modern method use revealed substantial underestimation of traditional method use, particularly in DRC and Ghana. These findings underscore the need to revise the current framing of questions and estimation approaches to improve the accuracy of contraceptive use estimates. The findings also highlight the importance of taking into account concurrent use of traditional and modern methods, which is often ignored in family planning research.

## BACKGROUND

Contraceptive prevalence estimates are key indicators of the performance of family planning (FP) programs, and they also inform policies on gender equality, fertility, and maternal and child health (Bongaarts, Mauldin, and Phillips 1990; Darroch and Singh 2013). Large‐scale surveys such as the Demographic and Health Survey (DHS), Performance Monitoring for Action (PMA), and Multiple Indicator Cluster Survey (MICS) are major sources of data for estimating prevalence and assessing the dynamics of contraceptive use in developing countries, particularly in sub‐Saharan Africa (SSA). More specifically, data from these surveys are routinely used to calculate indicators such as modern and traditional contraceptive prevalence, unmet need for FP, demand for FP satisfied with modern methods, contraceptive discontinuation, and method switching. Despite the widespread reliance on data from these surveys for decision‐making, there is evidence that these surveys may be underestimating the prevalence of contraceptives, particularly traditional and fertility awareness‐based methods (Polis et al. 2021; Rossier, Senderowicz, and Soura 2014).

The few small‐scale studies that have modified existing approaches for collecting, analyzing, and reporting data on contraceptive use all report higher contraceptive prevalence estimates compared to large‐scale surveys such as DHS and PMA (Marston et al. 2017; Polis et al. 2021; Rossier, Senderowicz, and Soura 2014). For example, Rossier, Senderowicz, and Soura (2014), using data from the Health and Demographic Surveillance System in Ouagadougou (Burkina Faso), estimated the prevalence of lactational amenorrhea (LAM), standard days method (SDM), periodic abstinence, and withdrawal at 26 percent compared to the DHS estimate of 5.1 percent.

The apparent underestimation of contraceptive prevalence in surveys such as DHS, PMA, and MICS stems from current methodological approaches, including the framing and ordering of survey questions as well as the approach used to calculate contraceptive prevalence (Marston et al. 2017; Rossier, Senderowicz, and Soura 2014; Staveteig 2017). For example, the standard question for eliciting data on current contraceptive use in DHS and PMA is framed as follows: *“Are you or your partner currently doing something or using any method to delay or avoid getting pregnant?”* There is evidence that some survey respondents interpret this question as only pertaining to modern methods because of words like “method” and “using.” Such an interpretation is likely to result in the underreporting of traditional and fertility awareness‐based methods. Evidence from a follow‐up study of women in the 2014 Ghana DHS showed that 34 percent of the sample that responded “no” to this question were actually using a method (Staveteig 2017). Marston et al. (2017) posit that women who use calendar methods often normalize them as part of their reproductive life and may not consider them as “methods” worth mentioning when responding to questions on contraceptive use. There are also concerns about what the word “currently” (in the standard question about contraceptive use) means to survey respondents, as it is not precise about the period of contraceptive use under question (Anderson and Cleland 1984; Choi et al. 2019).

Furthermore, the standard approach for calculating contraceptive prevalence privileges modern contraceptive prevalence (mCP) over traditional methods prevalence (tCP), consequently resulting in the underestimation of traditional methods (Polis et al. 2021; Senderowicz 2020; Rossier and Corker 2017). In this regard, contraceptive users are classified as either modern or traditional method users based on the “most effective method” they currently use or ever used. Thus, a woman who reports using a traditional and modern method(s) in the same time period (e.g., in the last one month) is classified as a modern method user, on the basis that the latter is their “most effective” contraceptive method (Marston et al. 2017; Polis et al. 2021). One of the few studies that tested the effect of modifying this standard prevalence estimation approach found that about 23 percent of women using periodic abstinence in Ghana would have been classified as condom or emergency contraceptive (EC) users, even though these women may have been using periodic abstinence as their primary contraceptive method (Polis et al. 2021). The current estimation approach may therefore result in significant underestimation of tCP in settings where women routinely combine traditional and modern methods.

Although a few studies in SSA have tested and suggested approaches for improving the estimation of contraceptive prevalence and enhancing our understanding of contraceptive use dynamics (Marston et al. 2017; Polis et al. 2021; Rossier, Senderowicz, and Soura 2014; Staveteig 2017), important gaps remain. First, all the previous studies had a limited geographic scope—that is, they were limited to a single country or setting within one country. The approaches suggested in these studies for improving contraceptive measurement have therefore not been tested across countries. Second, these studies did not address all the major methodological concerns affecting the estimation of contraceptive prevalence. For instance, while Polis et al. (2021), acknowledged that asking only respondents who answered “yes” to the question; *“Are you or your partner currently doing something or using any method to delay or avoid getting pregnant?”* could lead to the underreporting of non‐modern methods, their study did not include follow‐up method‐by‐method questions to women who responded “no” to this question, to ascertain whether they were using any method(s).

To improve the measurement of contraceptive prevalence and enhance our understanding of the dynamics of contraceptive use in SSA, we examined the effect of key survey methodological innovations on the estimation of mCP and tCP using comparative cross‐country data from the study, *“Re‐Examining Traditional Method Use”* (TEAM‐UP).^1^ We addressed two research questions: (1) Do modifications to standard survey approaches (used in national surveys such as DHS and PMA) result in different estimates of traditional and modern contraceptive use? (2) What modification(s) elicit different information from national surveys (such as DHS and PMA)? Ultimately, our aim in this paper is to stimulate further discussion and innovations in survey designs to better reflect the realities of what people do to prevent pregnancy.

## METHODS

### About the Parent Study: TEAM‐UP

The TEAM‐UP project was designed to measure the prevalence of modern and traditional methods and to understand women and men's motivations for using these methods. The project collected both quantitative and qualitative data in urban and rural sites in four SSA countries—the Democratic Republic of Congo (DRC), Nigeria, Ghana, and Kenya. Detailed descriptions of the TEAM‐UP methodology and data are available in Alhassan et al. (2026). Data from TEAM‐UP provides a unique opportunity to test the effect of key methodological innovations on the prevalence of traditional and modern methods. The data also provides an opportunity to analyze and compare the motivations and experiences of women and men using traditional and modern contraceptives in diverse settings in SSA.

### Study Setting

The TEAM‐UP study countries (DRC, Nigeria, Ghana, and Kenya) reflect the diversity in contraceptive prevalence and use patterns in SSA. The study sites were intentionally selected from areas (DHS or PMA regions) with contrasting contraceptive prevalence levels, ensuring that both high and low prevalence of traditional and modern methods were represented. Within each country, data were collected in two sites (one rural and one urban) to allow for comparisons of the effect of the methodological innovations on contraceptive use across different countries and contexts. In DRC, data were collected in two provinces: Mai‐Ndombe (rural site) and Kinshasa (urban site). Estimates from the 2014 DRC DHS show a higher tCP (15.2 percent) in Mai‐Ndombe than mCP (9.5 percent). More recent PMA data from 2018 for Kinshasa found that Kinshasa's tCP was similar to that of Mai‐Ndombe (16.2 percent), but its mCP was higher (25.3 percent) (Tulane University School of Public Health et al. 2018). When considered together, tCP in the two DRC sites is among the highest in the SSA region. Data collection in Nigeria was conducted in Adamawa (rural site) and Lagos (urban site) states. According to the 2018 Nigeria DHS, both modern (26.7 percent) and traditional (14.3 percent) contraceptive prevalence in Lagos was significantly higher than in Adamawa state at 17.9 percent and 5.9 percent, respectively (National Population Commission & ICF 2019).

Although the total fertility rate in Ghana and Kenya is much lower than in DRC and Nigeria, contraceptive prevalence in the two countries differs substantially. In Ghana, TEAM‐UP collected data in rural Greater Accra and urban Ashanti regions. According to the Ghana 2022 DHS, mCP in Greater Accra, which was estimated at 19 percent, was higher than tCP (6.5 percent) (Ghana Statistical Service(GSS) and ICF 2024). Traditional contraceptive prevalence (6.2 percent) in Ashanti is similar to that in Greater Accra, while mCP in Ashanti (15.5 percent) was lower than in Greater Accra. In Kenya, TEAM‐UP data were collected in Makueni (rural site) and Mombasa (urban site) counties. According to previous estimates from DHS and PMA, of all the eight study sites, the Kenya sites had the highest mCP (29.7 percent) in Mombasa and 41.8 percent in Makueni (KNBS and ICF 2023). The prevalence of traditional methods in Makueni (6.2 percent) was similar to the Ghana sites, whereas tCP in Mombasa (3.2 percent) was lower than all the other sites.

### Sample

In this paper, we analyzed data from the main TEAM‐UP survey conducted among women aged 15–49 years. The survey was conducted between September 2022 and March 2023. A multistage sampling technique was deployed to draw the study sample. In the first stage, a gridded population sampling technique was employed to generate the primary sample frames (clusters), without stratification. Thus, study sites were divided into clusters based on gridded population data of each site, using ArcGIS 10.6 parameters and the Gridsample algorithm in the R statistical package (Thomson et al., 2020). Geopode shapefiles of each site were used for selection in ArcGIS. Each cluster comprised between 30 and 60 households, depending on site complexity. A total of 271 clusters were randomly selected across the four countries, comprising 61 clusters in DRC, 60 in Nigeria, 47 in Ghana, and 103 in Kenya.

The second stage of sampling involved a complete household listing in each cluster to constitute the sample frame for the household selection. Between 30 and 60 households, depending on site characteristics, were randomly selected in each cluster for the household interviews. A complete list of household members was generated from interviewing household heads or other adult members capable of responding to the household questionnaire. The household questionnaire included information about the age, sex, and residence status (usual resident or visitor) of each household member. All eligible respondents (i.e., women aged 15–49 years who were usual residents of the selected household) were then interviewed using the women's questionnaire. A total of 13,633 interviews with eligible women across the four countries were completed. The analytic sample in this paper was restricted to 9,075 women who were either in union (married or living with a partner) or sexually active and not in union.

### Classifying Contraceptive Methods

Contraceptive methods are commonly categorized into traditional and modern methods, though there is no consensus on definitions for these two categories. As a result, a key methodological challenge to accurately estimating and comparing modern and traditional contraceptive prevalence is the lack of uniformity in the classification of specific contraceptives as either traditional or modern (Austad et al. 2016; Festin et al. 2016; Hubacher and Trussell 2015). For example, DHS defines traditional methods as periodic abstinence, withdrawal, and country‐specific folkloric methods such as herbs, amulets, and gris‐gris, while the World Health Organisation (WHO) restricts traditional methods to only periodic abstinence and withdrawal (Croft et al. 2023; Festin et al. 2016). In this paper, we use the stricter classification of traditional methods as withdrawal and periodic abstinence, as these two methods have proven efficacy (even if less than most modern methods), making them of greater relevance to FP programs. Although we excluded folkloric methods from the classification of traditional methods, it is worth noting that TEAM‐UP collected data on these methods but found their overall prevalence to be low (1.5 percent across all sites). The contraceptives classified as modern methods in this paper were female sterilization, male sterilization, intrauterine device (IUD), implants, injectables, pills, male condom, female condom, EC, basal body temperature, cervical mucus, SDM, and LAM.

### Methodological Innovations for Improving Contraceptive Prevalence Estimates

The aim of this paper was to assess how key survey methodological innovations affect the estimation of contraceptive prevalence. These methodological innovations included:

- *Revising the wording of questions on contraceptive use*: The TEAM‐UP survey revised the wording of key standard questions used to elicit data on contraceptive use in large‐scale surveys such as DHS and PMA. Specifically, questions on contraceptive use in the TEAM‐UP survey omitted the words “method” and “using” as these have been shown to prime respondents to focus on modern methods in their responses. For example, the DHS question *“Are you or your partner currently doing something or using any method to delay or avoid getting pregnant?”* was reworded as follows: *“Between now and the last one month, did you or your partner do anything or try in any way to delay or avoid getting pregnant?”* In addition, TEAM‐UP replaced the word “currently” in the standard DHS/PMA/MICS question with the phrase *“between now and the last one month”* to provide a more precise description of the period of contraceptive use under question, as the term “currently” is undefined and may be understood and interpreted inconsistently by respondents, especially for coitally dependent methods.
- *Follow‐up method‐by‐method questioning on contraceptive use among all respondents*: In contrast to surveys such as DHS and PMA, the TEAM‐UP survey asked all respondents questions on contraceptive use; that is, even respondents who initially said they were not using any method were still included in follow‐up questions about method use. This involved providing a detailed description of each method not spontaneously mentioned by respondents to ascertain whether respondents use them. It is important to mention that DHS has long adopted (including in the latest questionnaire modules) method‐by‐method questioning to elicit data on contraceptive knowledge (Croft et al. 2023). While DHS also uses method‐by‐method questioning to collect data on ever use of contraceptives, women or men who do not know about a method are not asked about ever using it. Such respondents are deemed to have never used that method. The limited use of method‐by‐method questioning in DHS and other national surveys makes TEAM‐UP one of the first to extend this questioning approach to elicit information about current contraceptive use, in addition to collecting data on knowledge and ever use of contraceptives. The aim of this innovation in TEAM‐UP was to reduce the apparent underreporting of traditional and fertility awareness‐based methods in surveys.
- *Differentiating methods as reported spontaneously or after follow‐up method‐by‐method questioning*: TEAM‐UP data specifies whether methods reported by respondents were spontaneously mentioned or reported after method‐by‐method questioning. Such a differentiation in the data enables an analysis of methods that are likely to be affected by underreporting in surveys that do not include method‐by‐method questioning.
- *Revising contraceptive prevalence estimation to account separately for concurrent use of modern and traditional methods*: In addition to estimating contraceptive prevalence using the standard “most effective” approach, we revised the estimation approach to also compute the percentage of women who report using both modern and traditional methods. The aim of the revised estimation was to determine the level of underestimation of traditional methods under the standard approach.

Additionally, TEAM‐UP also included experimental items on the strength of desire to delay pregnancy and perceived pregnancy risk, along with questions on other sexual practices used for pregnancy avoidance (e.g., non–penile–vaginal sexual practices) and on contraceptive use for sexually transmitted infections (STIs) prevention and menstrual management. Detailed analyses of these topics are beyond the scope of this paper, which focuses on improving contraceptive prevalence estimates, but the data are publicly available^2^ for further analysis (Alhassan et al. 2026).

### Analytic Strategy

We used frequencies and percentages to describe the demographic and socioeconomic characteristics (including age, marital status, educational attainment, wealth status, and place of residence) of the analytic sample. The analysis mainly focused on computing the percentage of women using modern and traditional contraceptives. We made these computations using the standard approach employed in surveys, such as DHS, and a revised estimation approach proposed by TEAM‐UP. In the standard estimation approach, we computed contraceptive prevalence based on the “most effective” method(s) used by women or couples to delay or avoid pregnancy. Thus, a woman who reported using both modern and traditional method(s) in the last one month of the survey was considered a modern method user, on the basis that the modern method was her most effective contraceptive method. In the revised estimation approach, we computed the percentage of women using modern and traditional methods regardless of efficacy. In this regard, women using both modern and traditional methods were categorized as concurrent modern and traditional users. Modern or traditional method users in the revised approach were women who relied exclusively on either of the two methods. Comparing prevalence estimates generated from the two approaches enabled us to quantify the level of underestimation/overestimation of traditional or modern methods in each site/country.

Given that TEAM‐UP data specifies whether contraceptive use was mentioned spontaneously by respondents or reported after method‐by‐method questioning, we calculated contraceptive prevalence separately for the methods mentioned before and after method‐by‐method questioning. This enabled us to assess the effect of method‐by‐method questioning on the reporting of modern and traditional methods in each country/site.

The data were weighted to account for unequal sampling probabilities and the complexity of the sampling design. All the analyses were conducted using STATA version 18.

## RESULTS

### Characteristics of the Study Sample

Out of the analytic sample of 9,075 women aged 15–49 years, 28 percent resided in DRC, 18 percent lived in Ghana, 28 percent in Kenya, and 25 percent in Nigeria. About three‐quarters (75.9 percent) of the Ghana sample lived in rural areas, while the majority of the samples in DRC, Kenya, and Nigeria resided in urban areas (Table 1). Most of the sample in each country had attained secondary or higher education (ranging from 50.6 percent to 66.9 percent), except in Kenya (35 percent). A majority of the sample in each country was in union (married or living with a partner). The percentage of the sample that had an intention to have a/another child ranged from 58.5 percent in Nigeria to 64.1 percent in Kenya. The majority of the sample in Ghana (57.4 percent), Kenya (59.4 percent), and Nigeria (56.6 percent) indicated that they would be unhappy or very unhappy if they were to get pregnant. Results for the prevalence of other sexual practices to avoid pregnancy (results not shown) ranged from 0.5 percent in Kenya to 4.2 percent in DRC.

**TABLE 1:** Demographic and socioeconomic characteristics of study sample

	DRC	Ghana	Kenya	Nigeria
	(*n* = 2,636)	(*n* = 1,660)	(*n* = 2,510)	(*n* = 2,269)
Background characteristics	(%)	(%)	(%)	(%)
Age (years)				
15–19	8.3	6.0	2.3	2.4
20–24	21.3	16.9	20.7	10.4
25–29	21.7	18.0	26.1	17.8
30–34	16.4	20.5	18.1	18.9
35–39	16.9	17.7	15.8	22.5
40–45	9.1	12.4	9.9	16.7
45–49	6.3	8.5	7.1	11.3
Place of residence				
Rural	26.7	75.9	22.2	22.8
Urban	73.3	24.1	77.8	77.2
Education				
No education	4.9	17.8	4.6	8.9
Primary	11.5	26.0	47.2	12.6
Secondary	66.9	50.6	34.8	51.1
Higher	16.7	5.6	13.4	27.4
Religion				
Catholic	12.8	3.4	15.9	4.2
Protestant	11.0	12.5	25.3	7.7
Charismatic	14.9	43.8	24.5	21.4
Other Christian	47.0	25.2	8.9	26.4
Islam	0.6	11.6	24.4	39.8
None/Other	13.7	3.5	1.0	0.5
Marital status				
Never in union	16.5	10.7	5.3	5.0
Currently married	62.9	63.7	85.0	88.0
Living together	19.0	24.7	7.0	5.0
Divorced/widowed	1.6	0.9	2.7	2.0
Fertility intention				
Have a/another child	63.2	60.4	64.1	58.5
No more	25.6	29.3	31.9	36.2
Undecided	11.1	10.3	4.0	5.3
Feelings about pregnancy				
Very happy	22.5	26.5	20.8	20.5
Somewhat happy	13.3	10.8	13.3	16.0
Not sure	13.9	4.2	5.4	6.2
Unhappy	23.1	23.1	24.8	29.4
Very unhappy	20.8	34.3	34.6	27.2
Don't Know	6.5	1.1	1.1	0.8
Site				
Kinshasa, DRC	84.2	‒	‒	‒
Mai‐Ndombe, DRC	15.8	‒	‒	‒
Ashanti, Ghana	‒	57.9	‒	‒
Greater Accra, Ghana	‒	42.1	‒	‒
Makueni, Kenya	‒	‒	27.4	‒
Mombasa, Kenya	‒	‒	72.6	‒
Lagos, Nigeria	‒	‒	‒	78.1
Adamawa, Nigeria	‒	‒	‒	21.9

SOURCE: TEAM‐UP Survey (2023).

### Effects of Methodological Innovations on the Estimation of Current Use of Traditional and Modern Contraceptives

Table 2 presents the results of the effect of follow‐up method‐by‐method questioning on the percentage of women using traditional and modern methods in the four study countries. The computation of tCP and mCP in Table 2 is based on the standard “most effective method” approach, which prioritizes modern over traditional methods. The results (Table 2) show that follow‐up method‐by‐method questioning substantially increases the prevalence of modern methods across all countries, especially in DRC, Ghana, and Nigeria. In DRC, follow‐up method‐by‐method questioning increased the percentage of women using modern methods from 20.1 percent to 32.2 percent. In Ghana and Nigeria, method‐by‐method questioning increased the prevalence of modern methods by about 5 and 4 percentage points, respectively. The lowest increase in modern method use following method‐by‐method questioning was in Kenya, where reported mCP before method‐by‐method questioning was already relatively high.

**TABLE 2:** Effect of follow‐up method‐by‐method questioning on estimates of traditional and modern contraceptive use (%) by country using the standard approach

	DRC		Ghana		Kenya		Nigeria	
	(*n* = 2,636)		(*n* = 1,660)		(*n* = 2,510)		(*n* = 2,269)	
	Before	After	Before	After	Before	After	Before	After
Contraceptive methods	(%)	(%)	(%)	(%)	(%)	(%)	(%)	(%)
Any modern	20.1	32.2	25.3	30.0	55.3	57.5	20.1	24.3
Traditional	9.0	11.4	4.7	5.9	3.9	4.7	12.1	13.8
Not using	70.9	56.4	70.0	64.1	40.8	37.8	67.8	61.9

SOURCE: TEAM‐UP Survey (2023).

Compared to modern methods, the results in Table 2 show little increase in the reporting of traditional methods following method‐by‐method questioning. The highest increase in traditional methods use after method‐by‐method questioning was in DRC (increasing by 2.4 percentage points), followed by Nigeria at 1.7 percentage points. Method‐by‐method questioning increased the prevalence of traditional methods in Ghana and Kenya by about one percentage point. The marginal increase in the use of traditional method(s) following method‐by‐method questioning may be attributable to the increased reporting of concurrent use of traditional and modern method(s) following method‐by‐method questioning. The results in Table 2 also show a substantial decrease in the percentage of women not using any contraceptive method after method‐by‐method questioning, ranging from a decline of 3 percentage points in Kenya to a high of 14.5 percentage points in DRC.

To disentangle concurrent traditional and modern method use from masking the effect of follow‐up method‐by‐method questioning on overall traditional contraceptive prevalence, we revised the estimation procedure to compute the percentage of women using modern and traditional methods to differentiate concurrent users of both method types. In this regard, women using traditional and modern methods concurrently in the one‐month period (otherwise classified as modern users in the standard estimation approach) were categorized separately as concurrent users. Thus, Table 3 shows the percentage of women using only modern methods, those using only traditional methods, and those using both methods concurrently, before and after method‐by‐method questioning.

**TABLE 3:** Effect of follow‐up method‐by‐method questioning on estimates of traditional and modern contraceptive use (%) in study countries using an estimation approach that accounts for concurrency

	DRC		Ghana		Kenya		Nigeria	
	(*n* = 2,636)		(*n* = 1,660)		(*n* = 2,510)		(*n* = 2,269)	
	Before	After	Before	After	Before	After	Before	After
Contraceptive methods	(%)	(%)	(%)	(%)	(%)	(%)	(%)	(%)
Modern	16.5	21.2	22.3	20.5	54.2	53.2	17.4	18.7
Traditional	9.0	11.4	4.7	5.9	3.9	4.7	12.1	13.8
Concurrent	3.6	11.0	3.0	9.5	1.1	4.3	2.7	5.6
Not using	70.9	56.4	70.0	64.1	40.8	37.8	67.8	61.9

NOTE: Totals may exceed 100%, as respondents can report use across multiple categories (modern, traditional, and/or concurrent).

SOURCE: TEAM‐UP Survey (2023).

The results in Table 3 confirmed that the increase in modern method use after method‐by‐method questioning (as observed in Table 2) was largely due to increased reporting of concurrent traditional and modern method(s) use. Specifically, method‐by‐method questioning increased the percentage of women reporting concurrent use of traditional and modern methods by 7.4 and 6.5 percentage points in DRC and Ghana, respectively. In Kenya, concurrent use of traditional and modern methods increased from 1.1 percent to 4.3 percent, while almost doubling in Nigeria after method‐by‐method questioning. Accounting for concurrent use of traditional and modern methods after method‐by‐method questioning shows a marginal decrease in modern contraceptive use in Ghana and Kenya, and a slight increase in Nigeria. DRC was the only country with a substantial increase in modern contraceptive use despite accounting for concurrent use of traditional and modern methods after method‐by‐method questioning, and this was largely driven by the patterns in Kinshasa.

The findings in Table 3 further demonstrate that the standard approach for computing contraceptive prevalence underestimates the prevalence of traditional method(s). For instance, the 11 percent and 9.5 percent of women using traditional and modern methods concurrently in DRC and Ghana, respectively, would have been classified as modern methods users under the standard estimation approach. Yet, these women may actually be using traditional contraceptives (withdrawal or periodic abstinence) as their primary methods while relying sporadically on modern methods such as condoms or EC. Accounting for concurrency means that the percentage of women using any traditional method in DRC and Ghana was 22.4 percent and 15.4 percent, respectively, not the 11.4 percent and 5.9 percent reported for the two countries under the standard estimation approach in Table 2.

For a more nuanced understanding of the effect of follow‐up method‐by‐method questioning, we examined the reporting of specific traditional and modern contraceptive methods before and after method‐by‐method questioning. The aim was to ascertain the specific traditional and modern methods that were likely to be affected by underreporting in the absence of follow‐up method‐by‐method questioning of survey respondents.

Table 4 shows the reporting of specific traditional and modern methods before and after method‐by‐method questioning in the DRC and Ghana sites, the two countries that had the greatest increase in reporting concurrent use. Overall, method‐by‐method questioning of respondents resulted in a substantially higher increase in the reporting of withdrawal and periodic abstinence compared to most of the modern methods. For example, method‐by‐method questioning resulted in a doubling of the prevalence of withdrawal and periodic abstinence in Kinshasa. Similarly, it increased the reporting of withdrawal from 7.2 percent to 13.3 percent in Accra (Ghana) while nearly tripling the prevalence of periodic abstinence in Kumasi (Ghana).

**TABLE 4:** Estimates of specific contraceptive methods before and after method‐by‐method questioning in DRC and Ghana sites

	DRC				Ghana			
	Kinshasa		Mai‐Ndombe		Ashanti		Accra	
	(*n* = 1453)		(*n* = 1183)		(*n* = 1105)		(*n* = 555)	
	Before	After	Before	After	Before	After	Before	After
Contraceptive methods	(%)	(%)	(%)	(%)	(%)	(%)	(%)	(%)
Traditional methods								
Withdrawal	6.4	13.7	13.2	19.4	2.2	4.8	7.2	13.3
Periodic abstinence	7.3	15.0	1.0	2.9	1.8	5.8	8.3	17.2
Modern methods								
Lactational amenorrhea	0.3	2.0	0.1	0.4	0.2	0.7	0.9	3.2
Standard days method	1.6	3.0	1.7	1.9	0.2	0.4	0.4	1.2
Cervical mucus	0.2	0.6	0.0	0.0	0.0	0.5	0.4	1.6
Basal body temperature	0.2	0.8	0.1	0.2	0.0	0.1	0.2	1.5
Emergency contraceptives	4.5	9.6	0.0	0.1	3.8	4.5	4.8	7.0
Female condom	0.1	0.5	0.4	1.2	0.0	0.0	0.0	0.0
Male condom	3.8	7.8	3.8	5.5	1.1	2.2	2.6	4.5
Pills	4.5	6.2	0.6	1.0	3.7	4.5	1.4	2.3
Implants	5.7	11.1	0.6	0.9	6.6	7.9	8.6	10.1
Injectables	3.5	6.3	0.7	1.2	10.2	11.7	6.9	9.2
Intrauterine device	0.0	0.3	0.0	0.0	0.3	0.3	0.1	0.4
Female sterilization	0.2	0.2	0.0	0.0	0.7	0.7	1.6	1.6

SOURCE: TEAM‐UP Survey (2023).

The results in Table 4 also showed substantial increases in the reported use of some modern methods following method‐by‐method questioning. Specifically, method‐by‐method questioning resulted in substantial increases in the reporting of LAM, EC, implants, and injectables in Kinshasa (DRC) and Accra (Ghana). For example, reporting of EC use increased from 4.5 percent to 9.6 percent in Kinshasa, while reporting of use of implants increased from 5.7 percent to 11.1 percent in the same site. In Accra, the reporting of LAM use increased from nearly 1 percent to 3 percent, while reporting of use of injectables increased from 6.9 percent to 9.2 percent.

Table 5 presents the results of the effect of method‐by‐method questioning on the reporting of specific traditional and modern methods in the Kenya and Nigeria sites. The effect of method‐by‐method questioning in reporting of withdrawal and periodic abstinence was similar in Mombasa (Kenya) and Adamawa (Nigeria), which are predominantly Muslim. The prevalence of withdrawal and periodic abstinence more than doubled after method‐by‐method questioning in Mombasa and Adamawa, although the levels were fairly low before and after method‐by‐method questioning. The increase in periodic abstinence following method‐by‐method questioning was modest in Makueni and Lagos.

**TABLE 5:** Estimates of specific contraceptive methods before and after method‐by‐method questioning in Kenya and Nigeria sites

	Kenya				Nigeria			
	Makueni		Mombasa		Lagos		Adamawa	
	(*n* = 939)		(*n* = 1571)		(*n* = 1263)		(*n* = 1006)	
	Before	After	Before	After	Before	After	Before	After
Contraceptive methods	(%)	(%)	(%)	(%)	(%)	(%)	(%)	(%)
Traditional methods								
Withdrawal	3.0	4.1	2.1	4.8	15.7	19.7	2.1	4.5
Periodic abstinence	5.6	7.3	2.1	5.0	4.0	5.8	0.8	3.4
Modern methods								
Lactational amenorrhea	0.1	0.4	0.5	0.7	1.1	1.6	0.5	3.4
Standard days method	0.0	0.2	1.4	2.2	0.9	0.9	0.1	0.1
Cervical mucus	0.1	0.4	0.3	0.6	0.2	0.9	0.0	0.0
Basal body temperature	0.0	0.0	0.0	0.0	0.1	0.4	0.0	0.1
Emergency contraceptives	1.4	1.7	1.1	1.5	2.4	2.5	0.1	0.8
Female condom	0.0	0.0	0.0	0.0	0.0	0.0	0.0	0.0
Male condom	2.9	3.9	3.1	4.5	6.3	8.7	1.3	3.4
Pills	7.4	7.7	6.5	6.7	3.9	4.2	1.0	2.7
Implants	19.3	20.6	22.1	22.3	4.6	4.8	2.5	5.2
Injectables	29.1	31.0	17.0	17.5	4.8	5.4	3.6	6.9
Intrauterine device	2.0	2.1	2.5	2.6	0.9	1.2	0.1	0.2
Female sterilization	1.6	1.6	0.6	0.6	0.4	0.4	0.1	0.1

SOURCE: TEAM‐UP Survey (2023).

The effect of method‐by‐method questioning on the reporting of modern methods in Adamawa was substantial, though overall prevalence was low. For example, reported LAM use increased from 0.5 percent to 3.4 percent; male condoms increased from 1.3 percent to 3.4 percent; pills from 1 percent to about 3 percent; implants doubled from 2.5 percent to 5.2 percent; and injectables from 3.6 percent to 6.9 percent. Compared to Adamawa and Kinshasa, the proportional increase in the reporting of modern methods after method‐by‐method questioning was modest in the Lagos, Mombasa, and Makueni sites.

## DISCUSSION

In this paper, we examined the effect of methodological innovations on the estimation of traditional and modern methods used in select sites in DRC, Ghana, Kenya, and Nigeria. The innovations included: (a) revising the wording of questions on contraceptive use, (b) method‐by‐method questioning following spontaneous responses to questions on contraceptive use, (c) differentiating contraceptive methods reported spontaneously and those reported after method‐by‐method questioning, and (d) computing contraceptive prevalence based on the standard “most effective” approach, and a revised estimation approach that accounts separately for concurrent use of traditional and modern methods.

The results showed that the addition of method‐by‐method questioning in the TEAM‐UP survey increased the reporting of traditional methods. Analysis of the percentage of women using withdrawal or periodic abstinence after method‐by‐method questioning shows substantial increases in most of the sites. This finding is consistent with those of the study by Rossier, Senderowicz, and Soura (2014) in Ouagadougou (Burkina Faso), which found that adding follow‐up questions to survey instruments significantly increased the percentage of women using withdrawal and periodic abstinence among other natural contraceptive methods. There is evidence elsewhere in SSA to show that methods such as periodic abstinence are rarely mentioned spontaneously as contraceptives used by respondents in surveys or qualitative interviews (Marston et al., 2017). Women who use withdrawal and periodic abstinence may consider these methods as part of their normal sex life and not as “contraceptive methods” (Polis et al., 2021) and may not spontaneously mention them in surveys, but rather only if prompted. It is also possible that traditional method users initially interpreted the question on “delaying or avoiding pregnancy” as only pertaining to modern methods, despite TEAM‐UP's approach of avoiding the term “method.”

The current study further showed that standard approaches for estimating contraceptive prevalence substantially underestimate the prevalence of traditional method use, particularly concurrent use with modern methods. For instance, a substantial proportion of women using traditional and modern methods concurrently in DRC and Ghana, respectively, would be classified as modern method users on the basis of method efficacy. If all women who report using any traditional methods (alone or concurrently with modern methods) are considered, the prevalence of traditional method use in DRC would be almost double the proportion using traditional methods under the standard reporting approach. Similarly, the percentage of women using any traditional method(s) in Ghana (alone or concurrently with modern methods) would be almost triple the proportion using traditional methods under the standard reporting approach. Our findings from Ghana corroborate results from a previous study in the country, which showed an increase in the prevalence of periodic abstinence based on all reported users of the method compared to reported use under the standard procedure for computing prevalence that prioritizes efficacy of the method (Polis et al. 2021). We note, however, that increases in reported traditional method use do not necessarily increase overall contraceptive prevalence, because many women report concurrent use with other methods. Our data thus add to existing evidence, which shows that the standard approach for calculating and reporting contraceptive prevalence (based on method efficacy) results in the underestimation of use of traditional methods, particularly in settings where concurrent use of traditional and modern methods is high.

A surprising finding of the current study was the increase in the reporting of modern methods following method‐by‐method questioning in some sites. This finding is in contrast to that of Rossier, Senderowicz, and Soura (2014), which did not show any increase in mCP in Ouagadougou despite including separate subquestions on every method. Reported modern methods use following method‐by‐method questioning, especially in Kinshasa, Accra, and Adamawa, increased for many modern methods, particularly EC, implants, and injectables. EC is thought to generally be under‐reported due to the more restrictive definitions of contraceptive use in surveys and the “emergency” phrasing around EC that may not resonate with planned or consistent use (Strong et al. 2025). Additionally, survey respondents who interpret ECs as abortifacients are not likely to report their use spontaneously because they do not consider them contraception or due to abortion stigma. Injectables, often a preferred method for covert use (Kushitor et al. 2022), may not be reported initially due to their discreet nature, with respondents possibly becoming more willing to disclose their use after gaining confidence in the survey process or interviewer during subsequent method‐by‐method probing. Women who adopted long‐acting methods in the more distant past may not report them as “current” or as used in the past month (Choi et al. 2019). Method‐by‐method questioning of all women, regardless of whether they spontaneously report using contraception, may thus increase the accuracy of reporting use of modern methods.

For DRC, and particularly in Kinshasa, the substantial and relatively higher increase in mCP observed after method‐by‐method probing was unexpected and initially raised concerns about data quality, especially given the large post‐probing increases in reporting of implants and injectables. However, the similarly high increases in reported traditional method use post‐probing in DRC, together with prior evidence on reporting inconsistency, may provide important context. A previous study that examined consistency in contraceptive reporting across six countries (India, DRC, Kenya, Nigeria, Niger, and Burkina Faso) found the highest inconsistency in DRC at follow‐up (Tsui et al. 2021). This suggests that the pattern of contraceptive use reporting in DRC is different from other settings in SSA, warranting further investigation to better understand the contextual drivers of contraceptive reporting in the country.

One of the key methodological innovations in the TEAM‐UP survey was the extension of follow‐up method‐by‐method questioning about current contraceptive use to women who spontaneously mentioned that they were not using any contraceptive(s). Polis et al. (2021) identified the lack of follow‐up questions to such women as a potential source of contraceptive use underreporting in surveys, but their study did not address this concern. The current study is, to the best of our knowledge, the first to explicitly test the effect of this innovation on contraceptive reporting across multiple SSA countries. The results revealed that follow‐up method‐by‐method questioning of such women substantially reduced the percentage of women who reported not using any contraceptive(s) in DRC, Ghana, and Nigeria, with a smaller reduction in Kenya. This finding is consistent with those from a follow‐up study to the 2014 Ghana DHS, which showed that many DHS survey respondents who indicated they were not using any method were actually using contraceptives at the time of the survey (Staveteig 2017).

## LIMITATIONS

A key limitation of this paper is that the TEAM‐UP data are not representative beyond the survey sites. Thus, the findings of this paper should not be interpreted as being representative of the four study countries—DRC, Ghana, Kenya, and Nigeria—or the regions in which they were conducted. A second limitation is that although the revised questionnaire wording, that is, specifying a time frame of “*between now and the last one month”* rather than asking about contraceptive use “*currently*,” was intended to test a more precise reporting period, it was not directly compared against the standard survey wording that asks about “methods.” As a result, we cannot determine whether this change affected reporting of contraceptive use or introduced differential reporting. However, initial estimates from the TEAM‐UP quantitative pilot were broadly consistent with comparable DHS data at the city or regional level, suggesting that the revised wording did not substantially influence reported method use. Future studies could more systematically compare these approaches to assess their equivalence. Moreover, while the findings of the study showed that method‐by‐method questioning increased the reporting of traditional and modern methods, there is the possibility that this approach may inadvertently encourage over‐reporting of contraceptive use, potentially due to social desirability effects brought about by the additional probing questions. Furthermore, the average time required to complete a TEAM‐UP questionnaire was higher than a standard DHS questionnaire—average time of 69 minutes to complete a TEAM‐UP interview compared to 30–60 minutes for DHS (Croft et al. 2023). Future research is required to address some of these limitations in order to provide more definitive data for strengthening current survey approaches and improving contraceptive use estimates.

## CONCLUSION

The findings of the current study have implications for FP survey designs, including the framing of questions, data collection, analyses, and reporting. The study found that including follow‐up method‐by‐method questioning in surveys increases the reporting of both traditional and modern methods. Extending follow‐up method‐by‐method questions to women who did not spontaneously report using contraception also reduced the percentage of women who reported not using any contraceptive. The results of this study point to the need to revise current survey approaches in order to capture and report important but nuanced and often ignored aspects of contraceptive use dynamics. Follow‐up method‐by‐method questioning may help to address misunderstandings and ambiguities around the wording of survey questions, elicit responses from respondents using contraceptives covertly, and those with difficulty recalling all their contraceptive use in the past. Incorporating this approach in survey designs requires extending follow‐up questions on contraceptive use to all survey respondents, with the potential downside of lengthening of questionnaires and time required to complete interviews. Further refining of measurement techniques that assess the levels and motivations for method concurrency, in particular, would allow surveys to more accurately capture and categorize contraceptive use, shedding light on these often overlooked aspects of contraceptive use dynamics.

## AUTHOR CONTRIBUTIONS

NA: Conceptualization, methodology, and original draft preparation. NJM: Conceptualisation, methodology, manuscript revision, supervision, and funding acquisition. JC: Conceptualisation, methodology, and manuscript revision. NDD: Conceptualisation, methodology, and manuscript revision. EC: Conceptualisation, manuscript revision, and supervision. FND: Conceptualisation, manuscript revision, and supervision. JE: Conceptualisation and manuscript revision. SP: Conceptualisation and manuscript revision. EO: Conceptualisation and data curation. MBP: Manuscript revision. JM: Manuscript revision. TM: Data curation. EMZ: Funding acquisition and manuscript revision.

## FUNDING INFORMATION

The TEAM‐UP project was supported by the Bill & Melinda Gates Foundation under grant INV‐006353.

## CONFLICT OF INTEREST STATEMENT

The authors declare that the research was conducted in the absence of any commercial or financial relationships that could be construed as a potential conflict of interest.

## ETHICAL STATEMENT

Ethical approval for both the pilot and main surveys was granted by review boards in the four countries. In Kenya, approval was granted by AMREF Ethics and Scientific Review Committee (Ref #: AMREF─ESRC P945/2021) and the National Commission for Science, Technology and Innovation (License #: NACOSTI/P/21/10530). Approval in Nigeria was granted by the National Health Research Ethics Committee of Nigeria (Approval #: NHREC/01/01/2007‐15/03/2021). In DRC, the study was approved by the Comité National d'Éthique de la Santé (National Committee of Health Ethics, Ministry of Health) under No.259/CNES/BN/PMMF/2021 du1 er /06/2021. In Ghana, the Ethics Committee for the Humanities (ECH) at the University of Ghana approved the study (Approval #: ECH 131.20‐21).

## Data Availability

The data analyzed for this paper can be accessed from the AFIDEP website after submitting a short request form via this link: https://afidep.org/publication/release‐of‐team‐up‐data/. The data package in this download contains a data file in STATA format, data use dictionary, and women's questionnaire.

## Footnotes

1 TEAM‐UP is the acronym coined to describe the consortium that implemented the project titled; Re‐Examining Traditional Method Use: Desperation or Innovation?

2 The main TEAM‐UP survey data [anonymized survey data stored in STATA format], women's questionnaire and data use dictionary are publicly available at https://afidep.org/publication/release‐of‐team‐up‐data/

## References

[sifp70054-bib-0001] Alhassan, Nurudeen , Jamaica Corker , Nyovani Janet Madise , Ernestina Coast , Naa Dodua Dodoo , Themba Mzembe , Jacques B. O. Emina , Elizabeth Omoluabi , F. Nii‐Amoo Dodoo , Saseendran Pallikadavath , Maame B. Peterson , John A. Mushomi , Funmilola M. OlaOlorun , and Eliya Msiyaphazi Zulu . 2026. “TEAM‐UP: Mixed‐Methods Data for Understanding Traditional and Modern Contraceptive Use Dynamics in Four Sub‐Saharan African Countries.” Studies in Family Planning, no. sifp.70046. 10.1111/sifp.70046. PMC13578833 41718497

[sifp70054-bib-0002] Anderson, John E. , and John G. Cleland . 1984. “The World Fertility Survey and Contraceptive Prevalence Surveys: A Comparison of Substantive Results.” Studies in Family Planning 15 (1): 1–13. 10.2307/1965479. 6701950

[sifp70054-bib-0003] Austad, Kirsten , Anita Chary , Alejandra Colom , Rodrigo Barillas , Danessa Luna , Cecilia Menjívar , Brent Metz , Amy Petrocy , Anne Ruch , and Peter Rohloff . 2016. “Fertility Awareness Methods Are Not Modern Contraceptives: Defining Contraception to Reflect Our Priorities.” Global Health, Science and Practice 4 (2): 342–345. 10.9745/GHSP-D-16-00044. 27353626 PMC4982257

[sifp70054-bib-0004] Bongaarts, John , W. Parker Mauldin , and James F. Phillips . 1990. “The Demographic Impact of Family Planning Programs.” Studies in Family Planning 21 (6): 299–310. 10.2307/1966918. 2075620

[sifp70054-bib-0005] Choi, Yoonjoung , Anoop Khanna , Linnea Zimmerman , Scott Radloff , Blake Zachary , and Danish Ahmad . 2019. “Reporting Sterilization as a Current Contraceptive Method among Sterilized Women: Lessons Learned from a Population with High Sterilization Rates, Rajasthan, India.” Contraception 99 (2): 131–136. 10.1016/j.contraception.2018.10.006. 30391289 PMC6367562

[sifp70054-bib-0006] Croft, Trevor N. , Courtney K. Allen , Blake W. Zachary , et al. 2023. “Guide to DHS Statistics.” https://www.dhsprogram.com/publications/publication‐dhsg1‐dhs‐questionnaires‐and‐manuals.cfm

[sifp70054-bib-0007] Darroch, Jacqueline E. , and Susheela Singh . 2013. “Trends in Contraceptive Need and Use in Developing Countries in 2003, 2008, and 2012: An Analysis of National Surveys.” Lancet 381 (9879): 1756–1762. 10.1016/S0140-6736(13)60597-8. 23683642

[sifp70054-bib-0008] Festin, Mario Philip R. , James Kiarie , Julie Solo , Jeffrey Spieler , Shawn Malarcher , Paul F. A. Van Look , and Marleen Temmerman . 2016. “Moving towards the Goals of FP2020 ‐ Classifying Contraceptives.” Contraception 94 (4): 289–294. 10.1016/j.contraception.2016.05.015. 27287693 PMC5032916

[sifp70054-bib-0010] Ghana Statistical Service (GSS) and ICF . 2024. “Ghana Demographic and Health Survey 2022.” https://ghana.unfpa.org/sites/default/files/pub‐pdf/ghana_dhs_2022_summary_report.pdf.

[sifp70054-bib-0013] Hubacher, David , and James Trussell . 2015. “A Definition of Modern Contraceptive Methods.” Contraception 92 (5): 420–421. 10.1016/j.contraception.2015.08.008. 26276245

[sifp70054-bib-0014] KNBS and ICF . 2023. “Kenya Demographic and Health Survey 2022: Key Indicator Report.” https://dhsprogram.com/pubs/pdf/PR143/PR143.pdf.

[sifp70054-bib-0016] Kushitor, Mawuli , Elizabeth G. Henry , Akua Danquah Obeng‐Dwamena , Martin Wiredu Agyekum , Caesar Agula , Theophilus Toprah , Iqbal Shah , and Ayaga A. Bawah . 2022. “Covert Contraceptive Use amongst the Urban Poor in Accra, Ghana: Experiences of Health Providers.” Reproductive Health 19 (1): 205. 10.1186/s12978-022-01516-5. 36333714 PMC9636747

[sifp70054-bib-0017] Marston, Cicely , Alicia Renedo , Gertrude Nsorma Nyaaba , Kazuyo Machiyama , Placide Tapsoba , and John Cleland . 2017. “Improving the Measurement of Fertility Regulation Practices: Findings from Qualitative Research in Ghana.” International Perspectives on Sexual and Reproductive Health 43 (3): 111–119. 10.1363/43e4517. 29553472

[sifp70054-bib-0019] National Population Commission (NPC) [Nigeria] and ICF . 2019. “Nigeria Demographic and Health Survey 2018.” https://dhsprogram.com/pubs/pdf/FR359/FR359.pdf.

[sifp70054-bib-0020] Polis, Chelsea B. , Easmon Otupiri , Suzanne O. Bell , and Roderick Larsen‐Reindorf . 2021. “Use of Fertility Awareness‐Based Methods for Pregnancy Prevention among Ghanaian Women: A Nationally Representative Cross‐Sectional Survey.” Global Health, Science and Practice 9 (2): 318–331. 10.9745/GHSP-D-20-00601. 34234024 PMC8324203

[sifp70054-bib-0022] Rossier, Clémentine , and Jamaica Corker . 2017. “Contemporary Use of Traditional Contraception in Sub‐Saharan Africa: Use of Traditional Contraception in Sub‐Saharan Africa.” Population and Development Review 43 (Suppl 1): 192–215. 10.1111/padr.12008. 29307923 PMC5749414

[sifp70054-bib-0023] Rossier, Clémentine , Leigh Senderowicz , and Abdramane Soura . 2014. “Do Natural Methods Count? Underreporting of Natural Contraception in Urban Burkina Faso.” Studies in Family Planning 45 (2): 171–182. 10.1111/j.1728-4465.2014.00383.x. 24931074

[sifp70054-bib-0024] Senderowicz, Leigh. 2020. “Contraceptive Autonomy: Conceptions and Measurement of a Novel Family Planning Indicator: Contraceptive Autonomy.” Studies in Family Planning 51 (2): 161–176. 10.1111/sifp.12114. 32358789

[sifp70054-bib-0025] Staveteig, Sarah. 2017. “Fear, Opposition, Ambivalence, and Omission: Results from a Follow‐up Study on Unmet Need for Family Planning in Ghana.” PloS ONE 12 (7): e0182076. 10.1371/journal.pone.0182076. 28759624 PMC5536298

[sifp70054-bib-0026] Strong, Joe , Ernestina Coast , Jamaica Corker , and Michelle Weinberger . 2025. “Capturing Emergency Contraceptive Pill Use: Critical Reflections on Measurement and Reporting.” Studies in Family Planning 56 (3): 470–481. 10.1111/sifp.70026. 40660800 PMC12501723

[sifp70054-bib-0027] Thomson, Dana R. , Dale A. Rhoda , Andrew J. Tatem , and Marcia C. Castro . 2020. “Gridded Population Survey Sampling: A Systematic Scoping Review of the Field and Strategic Research Agenda.” International Journal of Health Geographics 19 (1): 34. 10.1186/s12942-020-00230-4. 32907588 PMC7488014

[sifp70054-bib-0028] Tsui, Amy O. , Carolina Cardona , Varsha Srivatsan , Funmilola OlaOlorun , Elizabeth Omoluabi , Pierre Akilimali , Peter Gichangi , et al. 2021. “Is Client Reporting on Contraceptive Use Always Accurate? Measuring Consistency and Change with a Multicountry Study.” Studies in Family Planning 52 (3): 361–382. 10.1111/sifp.12172. 34383305

[sifp70054-bib-0029] Tulane University School of Public Health, University of Kinshasa School of Public Health and The Bill & Melinda Gates Institute for Population and Reproductive Health at The Johns Hopkins Bloomberg School of Public Health . 2018. “Performance Monitoring and Accountability 2020 (PMA2020) Survey Round 7, PMA2018/DRC‐R7‐Kinshasa Snapshot of Indicators.” https://www.pmadata.org/sites/default/files/2019‐12/PMA2020‐DRC‐Kinshasa‐R7‐FP‐SOI‐EN.pdf.

